# Antimicrobial regimens for the treatment of pan-drug-resistant *Acinetobacter baumannii* infections: some comments

**DOI:** 10.1128/aac.00077-26

**Published:** 2026-04-30

**Authors:** Salvatore Chirumbolo

**Affiliations:** 1 Department of Engineering for Innovation Medicine, University of Verona 19051, Verona, Italy; Houston Methodist Hospital and Weill Cornell Medical College, Houston, Texas, USA

**Keywords:** antibiotic resistance, *Acinetobacter baumannii*, bacteria, antimicrobial

## LETTER

The DESPAIR study compares the effectiveness of two triple-drug regimens against pan-drug-resistant *Acinetobacter baumannii* (PDR-AB) infections (1). While the study offers meaningful clinical insights, several flaws, biases, and limitations in its design and interpretation warrant consideration.

First, this is a retrospective analysis of prospectively collected data, inherently vulnerable to confounding and selection bias. The assignment of treatment regimens was nonrandomized and physician-dependent, potentially introducing unmeasured bias. Although inverse probability of treatment weighting (IPTW) and multivariable regression were used to adjust for confounders like SOFA scores, Charlson Comorbidity Index, and ICU status, residual confounding remains possible (1). For example, baseline differences in ICU admission rates (93% in group A vs 68% in group B, *P* = 0.002) suggest group A patients were more severely ill, yet they paradoxically had better outcomes, which may suggest unmeasured factors influenced regimen selection or outcomes (1).

Statistical power is also a concern. With only 83 patients in the primary analysis (60 in group A and 23 in group B), the study is underpowered for detecting moderate effect sizes, particularly in subgroup and multivariate analyses. Several estimates showed wide confidence intervals (e.g., adjusted HR for 28-day mortality with regimen B: 2.64 [0.99–7.02]), indicating imprecision. Small sample size also weakens the validity of the IPTW and regression models, which rely on large-sample properties to yield unbiased estimates (1).

Additionally, composite outcomes such as “clinical failure” may obscure interpretation. This outcome bundled disparate events (death, lack of improvement, toxicity-related withdrawal, persistent infection, and salvage therapy), which vary in clinical significance and etiology. For example, the significantly higher rate of drug withdrawal due to toxicity in group B (26% vs 1.7%) may be confounded by subjective clinician judgment and should perhaps be analyzed separately. Furthermore, while exclusion of toxicity from the composite outcome reportedly did not alter main conclusions, detailed sensitivity analysis data are relegated to supplementary tables, limiting transparency (1).

The lack of central microbiological testing introduces interlaboratory variability in pathogen identification and susceptibility testing, particularly for colistin, where broth microdilution was not universally used. Moreover, key pharmacodynamic factors such as minimum inhibitory concentration (MIC) values for tigecycline or sulbactam are not uniformly reported, limiting interpretation of observed efficacy.

The authors acknowledge some limitations but also speculate on mechanistic explanations, like pharmacodynamic synergy between meropenem and sulbactam, without confirming *in vitro* or *in vivo* synergy. While plausible, these hypotheses require direct experimental validation.

From a systems biology perspective, the study results have implications for forecasting antibiotic resistance dynamics. Using ordinary differential equations (ODEs), one might model the selective pressure imposed by each regimen on a mixed bacterial population.

**Fig 1:**
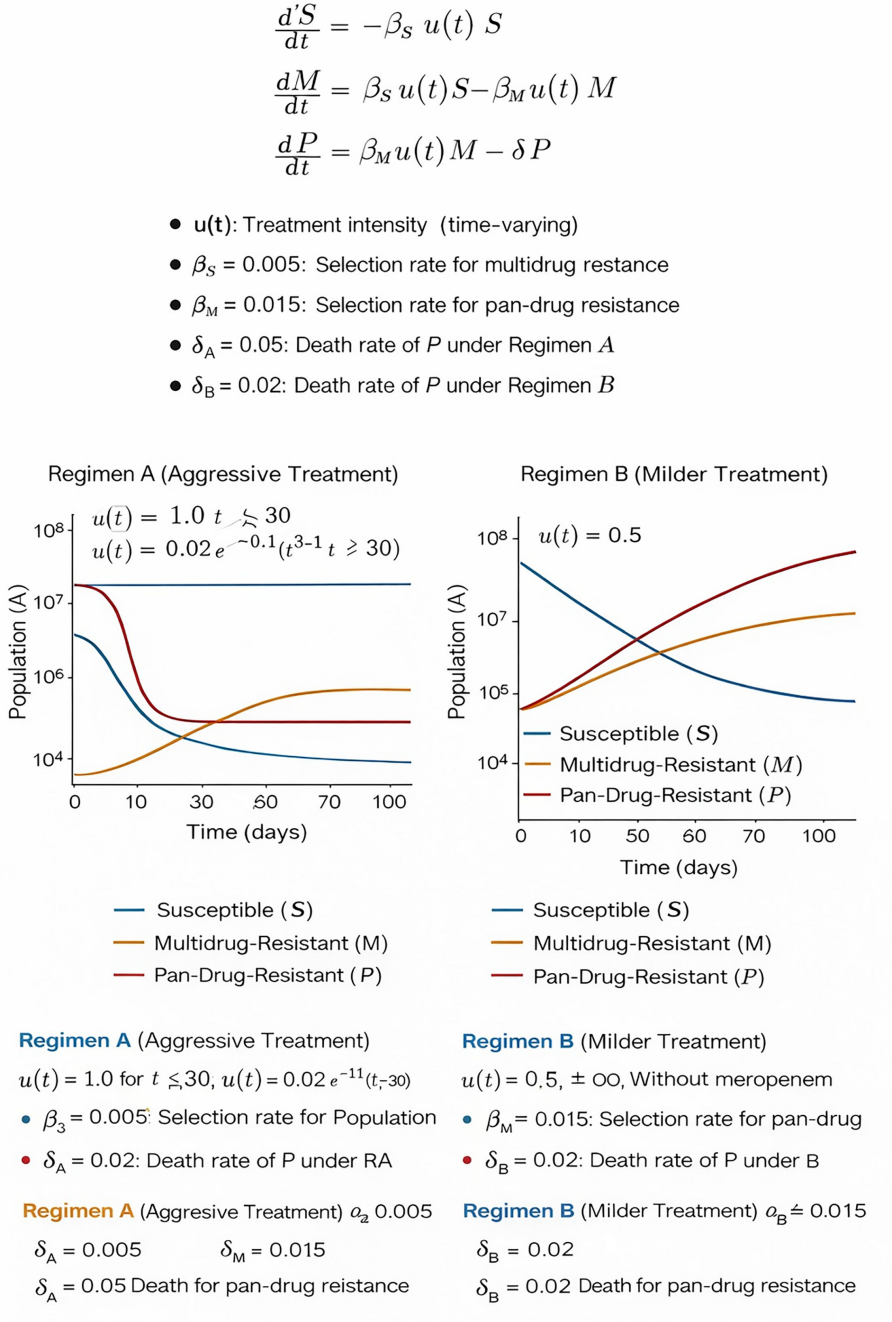
A systems biology model using ordinary differential equations (ODEs) to simulate the evolution of bacterial subpopulations, susceptible (S), multidrug-resistant (MDR) (M), and pan-drug-resistant (PDR) (P), under two antibiotic regimens. The equations model how treatment intensity u(t) drives selection from S to M to P, while δ represents the death rate of P. The left plot (Regimen A) depicts aggressive treatment with high initial intensity and a higher P-kill rate (δA = 0.05). Here, susceptible bacteria decline rapidly, MDR peaks early, and PDR levels eventually decrease. The right plot (Regimen B) shows a milder treatment (u(t) = 0.5) with a lower death rate (δB = 0.02); susceptible bacteria decline more slowly, while MDR and PDR populations steadily rise. The model highlights how aggressive regimens can suppress resistance more effectively in the short term, though both regimens risk driving resistance depending on selection pressure and microbial dynamics.

Suppose a system of ODEs describes the evolution of susceptible (*S*), multidrug-resistant (*M*), and pan-drug-resistant (*P*) subpopulations under treatment. A simplified model might be as follows:


$$\frac{dS}{dt}=-\beta_{s}u(t)S,\;\frac{dM}{dt}=\beta_{s}u(t)S-\beta_{M}u(t)M,\;\frac{dP}{dt}=\beta_{M}u(t)M-\delta P$$

where *u(t*) represents treatment intensity (higher for more aggressive combinations like regimen A), *β*_*S*_ and *β*_*M*_ are selection rates for resistance, and *δ* is a death rate for resistant pathogens. Regimen A may exert higher *δ* on P due to better synergy, effectively reducing the reservoir of PDR-AB. However, frequent use of meropenem in high-dose regimens could also accelerate selective pressure on carbapenem-resistant mutants. Incorporating pharmacokinetics/pharmacodynamics (PK/PD) into these models would refine predictions of resistance evolution and help balance efficacy against long-term resistance risks (Fig. 1).

In conclusion, while the DESPAIR study provides clinically relevant observational data favoring meropenem-based regimens, methodological limitations temper confidence in the findings. ODE-based modeling could extend the analysis by simulating how different regimens impact resistance trajectories over time, essential for sustainable antibiotic stewardship.
